# Poster Session II - A268 INCIDENCE OF INFLAMMATORY ARTHRITIS BEFORE AND AFTER INFLAMMATORY BOWEL DISEASE DIAGNOSIS: A POPULATION-BASED COHORT STUDY

**DOI:** 10.1093/jcag/gwaf042.268

**Published:** 2026-02-13

**Authors:** E Kuenzig, E Crowley, J Widdifield, E Benchimol, M Lam, V Jairath, S Rohekar, L Targownik, M Watson, R Berard

**Affiliations:** Western University, London, ON, Canada; Western University, London, ON, Canada; University of Toronto, Toronto, ON, Canada; University of Toronto, Toronto, ON, Canada; ICES, Toronto, ON, Canada; Medicine, Western University, London, ON, Canada; Western University, London, ON, Canada; University of Toronto, Toronto, ON, Canada; Western University, London, ON, Canada; Western University, London, ON, Canada

## Abstract

**Background:**

Arthropathies are a common extra-intestinal manifestation of IBD, yet population-level data on the risk of inflammatory arthritis (IA) among individuals newly diagnosed with inflammatory bowel disease (IBD) is lacking.

**Aims:**

Describe the incidence of IA among incident IBD cases.

**Methods:**

We used population-based health administrative data from Ontario, Canada to identify all incident IBD cases diagnosed between April 1, 2003 and March 31, 2020 using previously validated age-specific algorithms. Among individuals in this inception cohort of IBD patients, we identified individuals diagnosed with IA either before or after their IBD diagnosis. IA diagnosis (rheumatoid arthritis, ankylosing spondylitis, other seronegative spondyloarthropathies, arthralgia, joint swelling) required ≥1 hospitalization or emergency department visit or ≥ 2 physician claims with an IA diagnosis code, with ≥1 claim made by a rheumatologist, internal medicine physician, pediatrician, or gastroenterologist. IA diagnoses could occur at any point during data availability (1991-2024). We calculated the time between the diagnoses of IA and IBD, categorizing time into the intervals outlined in the Figure. Age- and sex-standardized IA incidence rates were calculated within each interval. Data were stratified by IBD type, age at IBD diagnosis (<18y, 18 to 64y, ≥65y), and sex.

**Results:**

We identified 56,776 individuals with incident IBD; 11,814 (20.8%) had an IA diagnosis. Characteristics of individuals with and without IA are described in the Table. The incidence of IA peaked in the 6 months prior to IBD diagnosis (23.1 (95% CI 18.6 to 28.4) per 1000 person-years), then gradually decreased in the time following IBD diagnosis. This pattern was consistent in all subgroups (Figure).

**Conclusions:**

This is the first population-based study to describe the incidence of IA in an inception cohort of people with IBD. The peak in IA diagnosis around the time of IBD diagnosis indicates a need for integrated multidisciplinary care models to facilitate patient access to both gastroenterology and rheumatology care. Future research will investigate the implications of these co-occurring diagnoses on health services utilization and direct healthcare costs.

A268 Table 1: Characteristics of patients with and without IA

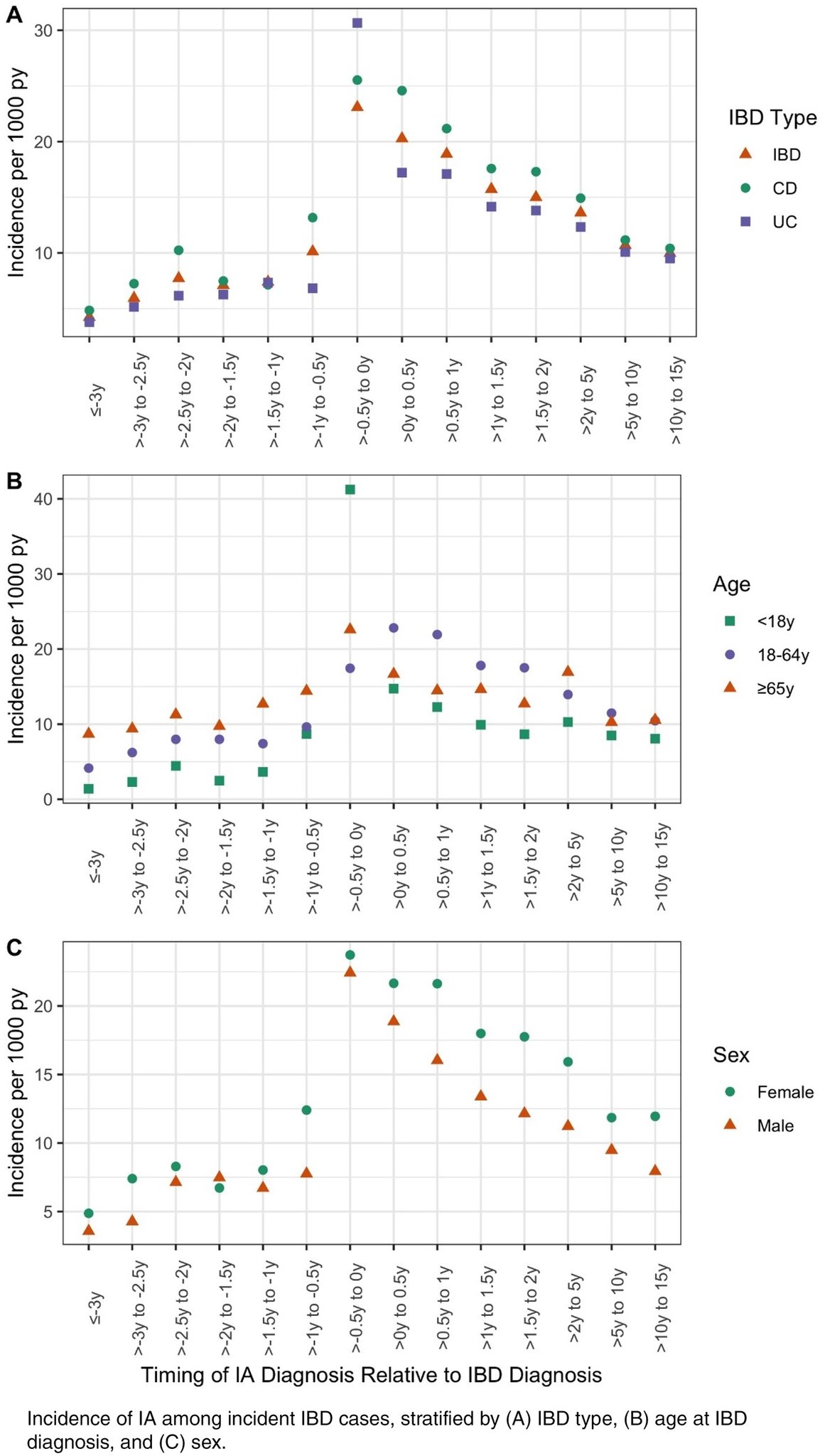

Incidence of IA among incident IBD cases, stratified by (A) IBD type, (B) age at IBD diagnosis, and (C) sex.

**Funding Agencies:**

CCC

**Table:** 

Characteristic	Ever diagnosed with IA (n = 11,814)	Never diagnosed with IA (n = 44,962)
Age at IBD diagnosis (years), mean (sd)	44.6 (18.5)	38.8 (19.1)
Female, n (%)	6818 (57.7)	21,942 (48.8)
Crohn’s disease, n (%)	5783 (49.0)	19,776 (44.0)
Ulcerative colitis, n (%)	5576 (47.2)	23,273 (51.8)
IBD-unclassiable, n (%)	455 (3.9)	1913 (4.3)
Rural residence at IBD diagnosis, n (%)	1569 (13.3)	4862 (10.8)

